# eXNVerify: coverage analysis for long and short-read sequencing data in clinical context

**DOI:** 10.12688/f1000research.121775.1

**Published:** 2022-06-13

**Authors:** Sebastian Porębski, Tomasz Stokowy

**Affiliations:** 1 Department of Cybernetics, Nanotechnology and Data Processing, Silesian University of Technology, Gliwice, Poland; 2 Department of Clinical Science, University of Bergen, Bergen, Norway

**Keywords:** long-read technology, whole genome sequencing, single nucleotide variants, sequencing coverage

## Abstract

Accurate identification of genetic variants to a large extent is based on the type of experimental technology, quality of the material and coverage of sequencing data obtained. The latter, coverage quality, highly influences variant calling accuracy and final diagnosis. Our motivation was to create a tool that will evaluate genome coverage and accelerate the introduction of long-read sequencing to medical diagnostics and clinical practice. The implementation was guided by the ease of use of the tool by users who are not proficient in using complex software. A Docker container is perfect for this purpose. Using Docker’s advantages (flexibility, mobility and ease of use of the proposed tools), we created eXNVerify. This is a tool for inspection of clinical data in the context of pathogenic variants search. The tool calculates clinical depth coverage (CDC) – a measure of coverage which we introduce to evaluate loci with pathogenic germline and somatic variants reported in ClinVar. The tool additionally provides visualization options for user-defined genes of interest. Finally, we present examples of BRCA1, TP53, CFTR application and results of a test conducted in the Extensive Sequence Dataset of Gold-Standard Samples for Benchmarking and Development. eXNVerify improves the diagnostic process of patients related to important genetic diseases and facilitates the assessment of genetic samples by diagnosticians. The use of Docker allows to run an analysis package and does not require any special technical preparation. Detailed examples are included in the GitHub project documentation and the package can be downloaded directly from DockerHub using the command: docker pull porebskis/exnverify:1.0.

## Introduction

Accurate identification of clinically relevant genomic variants strictly depends on sequencing coverage of sequencing data. Long-read (LR) sequencing covers a higher percentage of the human genome than short-read (SR) sequencing and results in more stable coverage ^1^. Consequently, single nucleotide, indel ^2^, and structural variants ^3^ are detected more accurately. Recent benchmarks evaluate the accuracy, precision, and recall of variant calling in long-read genome data ^4^; however, the introduction of new findings in the clinical and diagnostic setting requires more time. To accelerate the development of clinical genomics we present eXNVerify (named from “exon and single nucleotide variant verification”), a standalone tool that evaluates and visualizes genome coverage in a clinical context. While the available software approaches can analyze the sequencing data, none of them focuses on evaluating single nucleotide variant (SNV) coverage in the context of diagnostic procedures. This gap is filled by eXNVerify. The comprehensive quality control of medically relevant genes can be now adjusted to the diagnostic procedure. Moreover, the tool helps to verify the sequencing sample in terms of coverage of selected genes or to evaluate the overall genome/exome in terms of variant coverage.

## Methods

### Operation

eXNVerify consists of two procedures prepared in Python 3.8 with utilization of well-known numerical data-related libraries: numpy, pandas and matplotlib. Hence, for proper utilization of our tool, the user needs to install above packages in their Python distribution on their computer system. Source codes can be executed with the python command on Windows or Unix systems. However, we decided to publish a ready-to-go Docker container that includes all dependencies. If the potential user chooses the container, only the Docker application needs to be prepared and then our container can be pulled from the DockerHub repository with one line command: docker pull porebskis/exnverify:1.0.

### Implementation

The eXNVerify tool is designed to run clinically relevant coverage analysis for SR and LR data, providing integration with the ClinVar database. The software is designed as two standalone procedures: geneCoverage and snvScore. The primary input file for both procedures is the coverage record (BED format) obtained from processing BAM files. To create an input file, the user can use dependencies such as bedtools ^5^, mosdepth ^6^, or samtools ^7^ (see our GitHub documentation).

**Figure 1.**
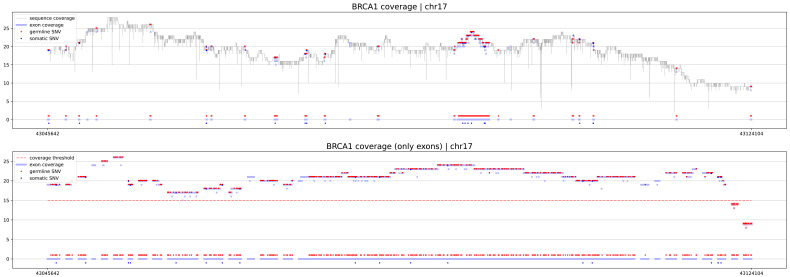
BRCA1 coverage for sample PacBio Long Read. The upper panel demonstrates the distribution of coverage in the region of the gene (exons and introns). The lower panel depicts coverage of exons. X-axis is a genomic locus, specified by the user. Dots highlight positions where pathogenic germline (red) and somatic (dark blue) ClinVar variants are located. If coverage of exons is lower than the threshold specified by the user, they are highlighted by the software in red color.

**Figure 2.**
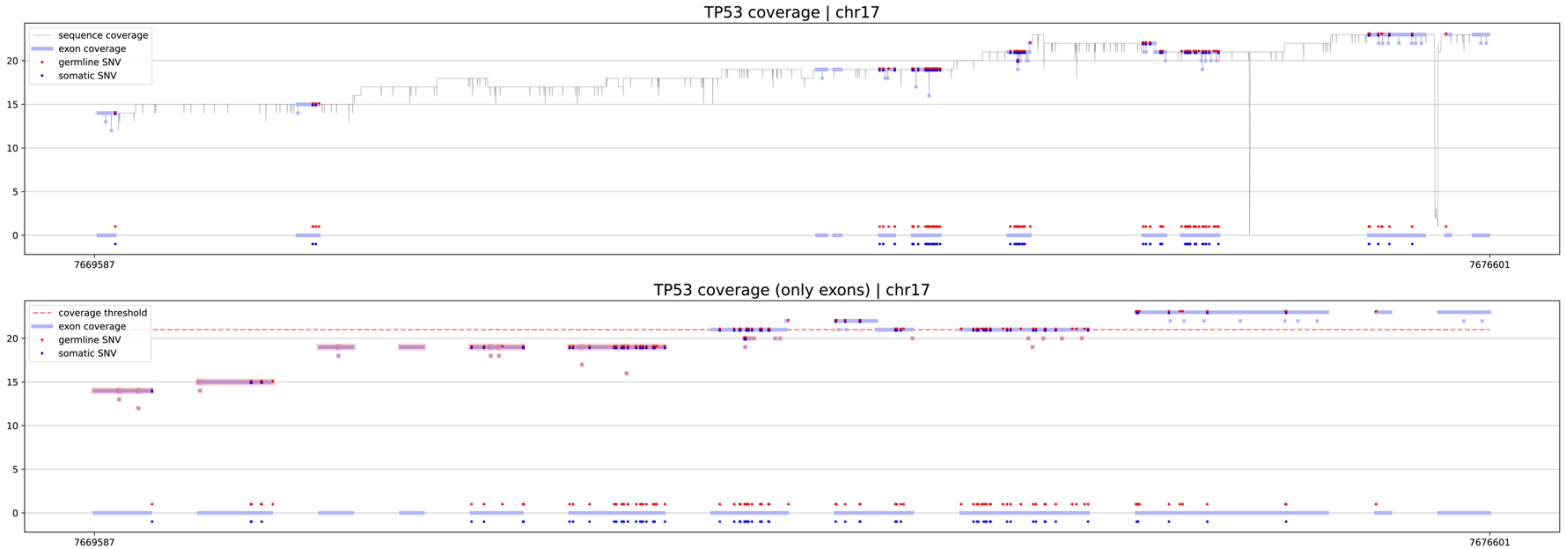
TP53 coverage for PacBio Long Read sample. Coverage of more than 40% of the gene did not reach expected 20x coverage. In such case diagnostic lab should consider optimization of the sequencing protocol, especially in exons 1, 2, 5 and 6, which include germline and somatic pathogenic variants.

**Figure 3.**
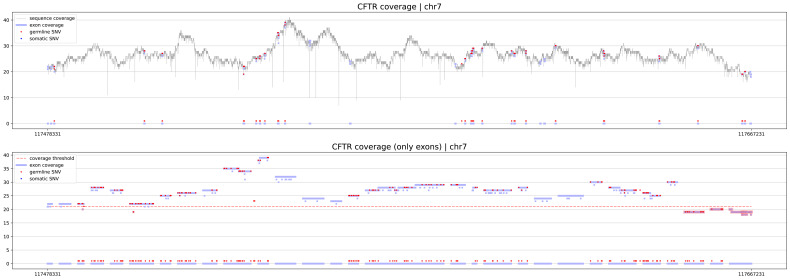
CFTR coverage for PacBio Long Read sample. eXNVerify scales visualization to correctly illustrate genes with large number of exons. In this case, CFTR gene, responsible for development of cystic fibrosis consist of 27 exomes.

The first procedure is geneCoverage. According to the location of exons of the selected gene, it presents coverage in a graphical form (coverages of exons are light blue fragments in Figure 1, Figure 2 and Figure 3) with the location of pathogenic SNVs. Germline and somatic variants are shown as red and dark blue dots, respectively. Moreover, geneCoverage counts the coverage of these SNVs and summarizes the results in a tabular form. The geneCoverage script, in addition to the exon list, pathogenic germline, and pathogenic somatic SNV list, also takes the names of the genes and the coverage threshold as a parameter. The latter is set to evaluate the sample if the gene-related variants are sufficiently covered. Thus, geneCoverage reports the percentage of SNV covered for a given gene and includes insufficient coverage in the generated figure. That is, specific exons that are poorly covered (which may contain key variants) are highlighted in red (see Figure 1, Figure 2 and Figure 3). This design helps to evaluate the reliability of the data before and after specific variant calling. Importantly, it is possible to prepare their own reference files with desired exon regions and SNV positions by following the examples provided in the referenced GitHub repository ( *Underlying data* ^8^).

An additional element of eXNVerify that focuses on the overall evaluation of sequence coverage is snvScore. It is used to check the coverage of all SNVs downloaded from the ClinVar database. The snvScore script checks variant coverage by all chromosomes and provides basic statistics. Finally, snvScore calculates a proposed measure of variant coverage, called clinical depth coverage (CDC), calculated as:


$${\text{CDC}}^{\left(t\right)}=\underset{m\in {\mathbb{N}}_{+}}{\arg\min}\sum_{i=1}^{N^{\left(t\right)}}\mathrm{sgn}\left[C\left(v_{i}^{\left(t\right)}\right)-m\right],\;t=\left\{ \begin{array}{l} g\;\text{for}\;\text{germline}\\ s\;\text{for}\;\text{somatic}, \end{array}\right.\#(1)$$

where $C(v_{i}^{\left(t\right)})$ is the coverage of the *i*-th SNV and *N* ^( *t*)^ is the number of all referenced variants. Germline and somatic variants are analyzed separately; hence *t* equals g or s for germline and somatic, respectively. We created examples for highly relevant genes for medical genetics and cancer genomics (see Supplementary Files 1 and 2, *Extended data* ^8^).

The sample BED file contains exclusive fragments of a gene sequence. Each fragment is related to one value of coverage. An exome reference list is also a BED format file, but it is different since it contains the location of all exons. It means that each row usually expresses a large fragment of sequence. Therefore, one fragment may be covered in a different number of reads. During the implementation, the task was to locate all fragments in the sample BED file that are in one exon fragment from the reference BED. In this way, coverages in one exon fragment are extracted. Next, if SNV location is available, it is possible to extract coverage information in formerly extracted exon coverage information. Coverage of SNVs is also presented graphically. Moreover, SNV coverage information is aggregated and summarized in table form in a report file. These operations are the core of the geneCoverage procedure.

The second procedure, snvScore explores the whole genome/exome and extracts coverage of all referenced SNVs coverage. Hence, snvScore requires a sample BED file and two ClinVar SNV tables. Exome reference and gene name are not necessary. The sample BED may be a large file, hence snvScore iteratively loads one-chromosome fragments, and extracts and aggregates information about SNV coverage. When finished, it reports CDC (1) for the whole sample with a via-chromosome table of germline and somatic pathogenic SNV coverage statistics. Supplementary File 1 ( *Extended data*) ^8^ provides a detailed example of snvScore execution.

## Results

eXNVerify is a new tool created to evaluate and visualize gene coverage in a clinical context. The tool consists of two methods implemented in Python: geneCoverage and snvScore. The first tool, geneCoverage looks for a gene (or multiple genes) of interest and evaluates it, integrating the coverage with the ClinVar pathogenic variant information. It demonstrates exons in a gene of interest, highlighting positions of pathogenic variants in the ClinVar database ( Figure 1, BRCA1 gene). The tool includes both germline pathogenic and somatic pathogenic SNVs. The tool is flexible and suitable for both oncology project ( Figure 2, TP53 gene) and rare disease projects ( Figure 3, CFTR gene). Processing the samples with the pandas and numpy libraries as well as visualization of the results with the matplotlib library is enough to provide intuitive support for the diagnostician. Moreover, geneCoverage indicates positions in which desired coverage has not been achieved and therefore variant analysis may lead to false negative/positive calls. The tool is suitable for LR and SR data providing novel insights and analysis options for all technologies used currently in clinical laboratories. To address the spectrum of technology-dependent coverage differences we present results for LR whole genome, SR whole genome and SR exome in Supplementary Figures 1 A, B, and C ( *Extended data*), ^8^; respectively. The second method, snvScore calculates coverage statistics for pathogenic variants, allowing the user to estimate the percentage of all SNVs that are covered above the defined threshold. Table 1 summarizes the essentials of its execution for test samples (Supplementary File 3, *Extended data* ^8^). 

**Table 1.** Results obtained for test samples. CDC: clinical depth coverage; s: somatic; g: germline.

Sample	Sample type	Expected mean coverage	CDC ^( *g*)^	CDC ^( *s*)^	Sample coverage median	% of germline variants covered above threshold	% of somatic variants covered above threshold
HG003	PacBio Long Read	21	20 ± 6	20 ± 5	22	81% (15x)	91% (15x)
HG003	Illumina WGS	20	24 ± 6	24 ± 6	24	94% (15x)	98% (15x)
HG003	Illumina Exome Agilent	100	141 ± 100	162 ± 108	18*	73% (100x)	84% (100x)

## Use cases

geneCoverage.py performs detailed verification of pathogenic germline and somatic SNVs for chosen gene(s) in a graphical from. Execution of the procedure require parameters in following order:


geneCoverage [-h]
                    SampleBED RefExomeBED SNVGermlineTXT SNVSomaticTXT
                    Threshold GeneName_s [GeneName_s ...]

positional arguments:
  SampleBED       Path to the mosdepth per-base BED output
  RefExomeBED     Path to the all exons BED file
  SNVGermlineTXT  Path to Clivar-generated table with pathogenic germline SNVs
  SNVSomaticTXT   Path to Clivar-generated table with pathogenic somatic SNVs
  Threshold       Coverage quality threshold
  GeneName_s      Gene name(s)

optional arguments:
  -h, --help      show this help message and exit

Exemplar execution of Docker container eXNVerify with geneCoverage.py on particular HG003 pacbio-hifi sample for verification of coverage of BRCA1 gene and all somatic and germline SNVs is as follows (code is split to couple of lines:

docker run -it --rm -v ~/hostpath/:/input \
-v ~/hostpath/:/output -v ~/hostpath/refs/:/refs \
porebskis/exnverify:1.0 ./geneCoverage.py \
input/HG003.pacbio-hifi.21x.haplotag.grch38.bam.per-base.bed \
refs/Exome_Reference_refined.bed refs/SNV_patho_germline.txt \
refs/SNV_patho_somatic.txt \
15 BRCA1


The crucial result of abovementioned procedure is graphical file with coverage analysis results of BRCA1 genome sequence (see Figure 1). More examples and results can be directly downloaded from 8.

snvScore.py analyses the whole genome sequence coverage and evaluate all pathogenic germline and somatic SNV coverage quality. Its execution needs to fit the procedure positional arguments as follows:


snvScore [-h] SampleBED SNVGermlineTXT SNVSomaticTXT [Threshold]

positional arguments:
  SampleBED       Path to the mosdepth per-base BED output
  SNVGermlineTXT  Path to Clivar-generated table with pathogenic germline SNVs
  SNVSomaticTXT   Path to Clivar-generated table with pathogenic somatic SNVs
  Threshold       SNV coverage quality threshold (optional, positive)

optional arguments:
  -h, --help      show this help message and exit


Exemplar snvScore.py execution within eXNVerify Docker container for sample coverage BED file is as follows:


docker run -it --rm -v ~/hostpath/:/input -v ~/hostpath/:/output \
-v ~/hostpath/refs/:/refs porebskis/exnverify:1.0./SNVScore.py \
input/HG003.pacbio-hifi.21x.haplotag.grch38.bam.per-base.bed \
refs/SNV_patho_germline.txt refs/SNV_patho_somatic.txt 15


The aim of snvScore.py is to prepare coverage analysis of all referenced SNVs in the tabular form ( Table 1). summarizes the results of snvScore execution of different genome sequence data. In the project documentation ^8^, the reader may find snvScore results in tabular via-chromosome qualitative results.

## Conclusions

The tool can be used to inspect structural variants observed in the sample, especially deletions and copy number changes. This approach can be helpful in a manual verification of structural variants, which is still a recommended practice in medical genetics ^9^.

Finally, eXNVerify gives insights into the sample’s usefulness in a hypothesis-free analysis of pathogenic variants. The proposed CDC measure provides a percentage of variants covered above the desired threshold in a specified case ( Table 1 and Supplementary File 3, *Extended data*, ^8^). This measure is useful for everyday laboratory practice to maintain and maximize the quality of experiments. Results of such analyses are provided in Table 1, which indicates the percentage of germline and somatic pathogenic variants specified above the desired threshold. It can also be observed that pathogenic variant coverage differs from median coverage and mean coverage of the sample. For the HG003 PacBio Long Read sample, clinical depth coverage equaled 20x, while global median coverage was 22x. A user of the software can also see that 81% of germline pathogenic variants were covered at least 15x.

We conclude that CDC measures and the percentage of variants covered above the threshold are useful for medical genetics and cancer diagnostics. In summary, our new tool introduces new, easily applicable options for medical genome analysis.

## Software availability

Ready-to-go Docker container can be pulled from https://hub.docker.com/r/porebskis/exnverify. Source code available from: https://github.com/porebskis/eXNVerify. Archived source code as at time of publication: https://doi.org/10.5281/zenodo.6541899

License: MIT

## Data availability

### Underlying data

Test samples were taken from the public repository provided by Google Cloud Storage. The only requirement for users to browse this repository is to have Google account. These data are released under CC-0 license and introduced by Baid *et al.*, 2020 ^4^. Instructions for accessing this public data can be found in Google Cloud Storage documentation. For user consideration, we provide the following public links to HG003 samples, generated with three different sequence technologies: PacBio Long Read (42.1 GB), Illumina WGS (38.9 GB), and Illumina Exome Agilent (8.4 GB)

### Extended data

GitHub: https://github.com/porebskis/eXNVerify/tree/main/suppdata This project contains the following extended data:

S1 Fig BRCA1 coverage for samples: A – PacBio Long Read, B – Illumina WGS, C – Illumina Exome Agilent. Detail description as for Fig 1.

S1 File Supplementary Data – exemplar use cases of eXNVerify with quantitative and graphical results

S2 File geneCoverage report for HG003 PacBio LR, Illumina WGS, Illumina Exome Agilent

S3 File snvScore report for HG003 PacBio LR, Illumina WGS, Illumina Exome Agilent
