# Supplementary material for: eXNVerify: coverage analysis for long and short-read sequencing data in clinical context
Source: F1000Res. 2022 Jun 13;11:645. [Version 1] doi: 10.12688/f1000research.121775.1 (PMC10762294; doi:10.12688/f1000research.121775.1)
Supplement: Peer review history

# Reviewer report: Reviewer response for version 1

**DOI:** 10.5256/f1000research.133674.r228027

**Published:** 2024-01-24

**Authors:** Wouter De Coster

**Affiliations:** 1 University of Antwerp, Antwerp, Belgium

The authors describe eXNVerify, a tool to assess the coverage in sequencing data. This is relevant, as coverage is a crucial parameter for variant detection and accuracy. The tool is well developed and the plots are mostly clear, but overall the scope of this tool is relatively narrow - focusing on one sample at a time. Please find my detailed comments below.

 In the introduction, the authors mention that long-read sequencing covers a higher percentage of the human genome and results in more stable coverage. While the stable coverage is probably mostly a feature of the absence of PCR amplification, I would mainly warrant some caution about the sentence following that statement: 'Consequently, single nucleotide, indel, and structural variants are detected more accurately'. I believe this statement to be incorrect, specifically in the case of short indels - as such variants are the dominant error mode of nanopore sequencing. While long reads enable alignment in highly repetitive regions, and thus variant calling there, some more nuance is warranted with regard to which variants are more accurately detected. I am a bit unsure if the focus on long-read sequencing in the introduction is relevant, as your tool appears to be broadly applicable.

 I am a bit confused by the bottom panels of Figures 1 and 3 and both panels in Figure 2. Do these plots contain two samples, with the blue lines of exon coverage at y=0? Why are variants shown twice, once in the plot at different heights and once at y=1/-1? 

 The legend of Figure 3 mentions that it consists of "27 exomes". which should presumably be 27 exons.

 Can the authors elaborate on what the coverage threshold of 15x was chosen for Figure 1, and 20x for Figure 2? I also think that the converge threshold line in Figure 2 is not at 20x but just above that.

 I think distributing your tool via docker might make it easier for some people, as installation becomes easier, but then requires that docker is already installed, which may not be the case. I would suggest that you include installation instructions (using pip or conda) for those users that cannot use docker. Regrettably, not all sysadmins will allow users to use docker. 

 I see in the GitHub documentation that the tool requires a 'Path to Clivar-generated table with pathogenic germline SNVs,' but it is not clear how that one needs to be generated. Example files are provided in the repository, which is highly appreciated, but it is unclear to which reference genome they correspond. It would however be most useful if your tool included a command to download such a table automatically from the clinvar database. The same argument could be made for the exome bed file. In addition, the authors recommend Mosdepth, which is an excellent choice, but I would also suggest including optimal parameters for this tool in your documentation, as it has various options. I see the SNVSomaticTXT is a required argument, but I can imagine that somatic variants are not necessarily relevant for everyone. Maybe that could be an optional input, and maybe a suitable default could already be set for the threshold. In addition, if users have many genes of interest, they will have to provide them all one by one on the command line, which is probably frustrating. 

 The example docker command for geneCoveray.py in the GitHub documentation misses a trailing slash '\', and thus yields a confusing error. It also appears the coverage bed file cannot be (b)gzip compressed? That is very unfortunate and something I would suggest adding.

 While not critical, I think it would be interesting if users could provide multiple samples as input (e.g. a trio), for comparison, rather than having to run the tool multiple times and open multiple separate plots.

 That said, after downloading all files, the tool ran as expected and the plots provided a useful representation of the coverage.

Are the conclusions about the tool and its performance adequately supported by the findings presented in the article?

Partly

Is the rationale for developing the new software tool clearly explained?

Yes

Is the description of the software tool technically sound?

Yes

Are sufficient details of the code, methods and analysis (if applicable) provided to allow replication of the software development and its use by others?

Yes

Is sufficient information provided to allow interpretation of the expected output datasets and any results generated using the tool?

Partly

Reviewer Expertise:

bioinformatics, data visualization, sequencing data analysis

I confirm that I have read this submission and believe that I have an appropriate level of expertise to confirm that it is of an acceptable scientific standard, however I have significant reservations, as outlined above.

# Reviewer report: Reviewer response for version 1

**DOI:** 10.5256/f1000research.133674.r228033

**Published:** 2024-01-02

**Authors:** Xueyi Dong

**Affiliations:** 1 The Walter and Eliza Hall Institute of Medical Research, Victoria, Australia

In this article, the authors introduced their software tool eXNVerify, which provides functions to visualize and check variant coverage of genes.

 Overall, the functions and usage of eXNVerify are introduced clearly in this article. The software tool shows good practical value in making it easier to investigate SNV coverage from sequencing data. The format of visualization that eXNVerify takes is also clear and helpful. However, I have several minor comments:

 1. In the exon-only coverage plots (Figure 1-3 bottom panels), the x-axis is scaled to skip the intron regions and highlight the exon regions. It helps show more details of SNVs in exon regions, especially for genes with lots of exons. However, the starting and ending point labels on the x-axis are misleading. It should be reflected on the plots of how the axis was scaled, such as including a scale bar of genomic positions.

 2. The software tool is provided as a docker image, which simplifies the installation procedure. However, some institution computers and/or servers don't support docker. Are alternative installation methods available?

Are the conclusions about the tool and its performance adequately supported by the findings presented in the article?

Yes

Is the rationale for developing the new software tool clearly explained?

Yes

Is the description of the software tool technically sound?

Yes

Are sufficient details of the code, methods and analysis (if applicable) provided to allow replication of the software development and its use by others?

Yes

Is sufficient information provided to allow interpretation of the expected output datasets and any results generated using the tool?

Yes

Reviewer Expertise:

bioinformatics

I confirm that I have read this submission and believe that I have an appropriate level of expertise to confirm that it is of an acceptable scientific standard.

# Reviewer report: Reviewer response for version 1

**DOI:** 10.5256/f1000research.133674.r140681

**Published:** 2022-06-27

**Authors:** Zebin Zhang

**Affiliations:** 1 Division of Population Genetics, Department of Zoology, Stockholm University, Stockholm, Sweden

Porębski S and Stokowy T provide a software, “eXNVerify,” which evaluates and visualizes genome and variations coverage for long and short reads sequencing data in the clinical context. This software is useful, especially for users who are not proficient in bioinformatics, as it is easy to install, use, and visualize results. It also filled the gap in evaluating the coverage of SNV in the context of diagnostic procedures.

 While much of the descriptions and results are clear, there are some minor concerns about this paper. These concerns are laid out below:

 1. The authors declare that their software can work on long and short reads sequencing data in the title. I believe this as the authors exhibited long-read results in the results section and short-read results in the supplementary section. However, in the abstract, the authors describe, “Our motivation was to create a tool that will evaluate genome coverage and accelerate the introduction of long-read sequencing to medical diagnostics and clinical practice,” without mentioning short-read sequencing. Is this implying that the result for the short read is not as reliable as the long read?

 2. Methods - operation section. The source codes seem only work on Windows and Linux systems. Because many people work on this system, especially those who are not proficient in bioinformatics or can’t access any servers. So my question is, does the eXNVerify work on the macOS system? If not, please explain why.

 3. The function of highlighting the insufficient coverage is pretty good, and the authors give some examples of the coverage thresholds 15x and 20x. Why did the authors choose those two values? Are these values arbitrary or selected based on some statistical consideration? Can the software give users a recommended value for the threshold?

 4. In fig 3 and 4, the threshold line of 20 doesn’t match the y-axis.

 5. Given the importance of de novo mutation in clinical, is that possible for authors to add another function of output the coverage of those variations?

 6. Mapping quality is also critical in variants calling, do the authors ever consider quality in variants evaluation and CDC calculation?

Are the conclusions about the tool and its performance adequately supported by the findings presented in the article?

Yes

Is the rationale for developing the new software tool clearly explained?

Yes

Is the description of the software tool technically sound?

Yes

Are sufficient details of the code, methods and analysis (if applicable) provided to allow replication of the software development and its use by others?

Yes

Is sufficient information provided to allow interpretation of the expected output datasets and any results generated using the tool?

Yes

Reviewer Expertise:

Bioinformatics

I confirm that I have read this submission and believe that I have an appropriate level of expertise to confirm that it is of an acceptable scientific standard.
